# A Scoping Review of Strategies to Increase Newcomer Family Access to Early Childhood Services

**DOI:** 10.1007/s11121-025-01867-y

**Published:** 2026-02-11

**Authors:** Elly Miles, Erin Doyle, Soumita Bose, Hamutal Bernstein

**Affiliations:** Urban Institute, 500 L’Enfant Plaza SW, 20024 Washington DC, USA

**Keywords:** Refugee, Immigrant, Early childhood, Child care, Home visiting, Early intervention, Access

## Abstract

Early childhood services can lay a critical foundation for refugee and immigrant children as they develop in new cultural contexts; however, these populations are underrepresented in a variety of early childhood programs such as early care and education, home visiting, and early intervention. This scoping review examines the strategies being implemented to increase newcomer families’ participation in services and parent preferences for early childhood services. A systematic search yielded 38 studies, 22 of which included strategies to increase access for newcomer families and 20 of which explored parent preferences around early childhood education. Identified strategies to promote access were categorized as program responsiveness (67%), outreach (57%), workforce responsiveness (57%), service delivery (52%), added supports (52%), social networks (43%), partnerships (43%), program policies (43%), or state and national policies (29%). Parents’ preferences for care were related to child academic and social-emotional development (80%), perceived quality (65%), type of care (65%), cultural responsivity and match (60%), and language (50%). Most studies focused on access or preferences related to early childhood programs or child care and early education broadly (89%), while fewer focused on strategies to increase access to home visiting or early intervention (11%). Additional research is needed to identify the strategies being utilized to promote access to home visiting and early intervention and to empirically test the relationship between identified strategies and improved access to these services.

**Supplementary Information:**

The online version contains supplementary material available at 10.1007/s11121-025-01867-y.

## Introduction

Publicly funded early childhood services (ECS) can lay a critical early foundation for refugee and immigrant children (RIC) as they grow and develop in new cultural contexts. A range of widely accessible ECS in the USA including subsidized child care, Head Start, state-funded preschool, home visiting, and early intervention together lay a strong foundation with impacts on child development, school readiness, social-emotional well-being, and long-term achievement and health (Hofferth & Collins, 2000; Mondi et al., 2021; Sabol & Chase-Lansdale, 2015; Vesely et al., 2013). These programs share an emphasis on whole family comprehensive supports, often including efforts to support parents in identifying and meeting their goals (e.g., employment and educational opportunities), connect families with essential community resources, and promote the academic and socioemotional development of children. However, data indicate that RIC are underrepresented in early childhood services for which they are eligible (Capps & Newland, 2015; Gallegos et al., 2021; Hardy et al., 2020; Matthews & Ewen, 2006; Park & Katsiaficas, 2019), and that families encounter an array of barriers to accessing public programs (Perreira et al., 2012). While literature has characterized a wide array of barriers, including family preferences and beliefs, that may drive disparities (Greenberg et al., 2016; Perreira et al., 2012; Seibel, 2021), less is known about the ways to reduce disparities in access.

### Newcomers: Definition and Experiences

Newcomer families, including refugees and immigrants, have migration-specific journeys and lived experiences that may differentially impact their health, well-being, and integration experiences in their host countries (Miles et al., 2019; Phillips & Robinson, 2015; Virupaksha et al., 2014). Many immigrant families come from low- and middle-income countries (World Migration Report, 2024), along with the vast majority of refugees (UNHCR, n.d.). Families in these contexts may be less likely to have accessed publicly available early childhood-focused services that focus on healthy development and family well-being (Bunn & Betancourt, 2022; Scharf et al., 2021). Refugees are forced to leave their home countries due to risk of persecution, conflict, or violence (UNHCR, n.d.), and across their migration experience, parents and children commonly experience wide-ranging stress exposures including trauma, exploitation, repeated displacements, separation from family, and exposure to material hardship and resource deprivation (Miles et al., 2019). While immigrants migrate from their countries of origin for a range of reasons, they may also be exposed to political violence and economic distress in their countries of origin (Paat, 2013; Rousseau & Drapeau, 2004). Many experience interpersonal violence or witness violence inflicted on family members pre-migration and detention and separation from family during their migration journeys (Scharpf et al., 2021). Together, these chronic and high-stress events are associated with high rates of psychological distress and post-traumatic stress disorder in adults (Scharf et al., 2021), worsening mental health in children, and strain on parent–child relationships (Miles et al., 2019; Sim et al., 2018). These experiences further signal a high likelihood that children have been exposed to stress that is “toxic” to their developing brains and bodies during vulnerable periods of development (Bucci et al., 2016).

These stressful and traumatic experiences are the backdrop against which many refugee and immigrant families contend with the cultural and linguistic challenges of acculturation (Hooper et al., 2016; Scharf et al., 2021; Schick et al., 2016). Children are profoundly impacted by these early traumatic experiences, with as many as 98% of young refugee children demonstrating delays or difficulties in multiple domains of development (Signorelli et al., 2017) and a high prevalence of emotional and behavioral health difficulties in resettled children (Fazel et al., 2012). Families experience the toll of these cumulative stress exposures while often experiencing social isolation and limited supports in resettlement (Cureton, 2020; Edwards et al., 2020; Scharf et al., 2021). Left unaddressed, exposure to toxic stress, including the structural, cultural, and interpersonal racism that resettled families may experience, is thought to account for poor physical and mental health across the lifespan, reduced achievement, and lost human potential (Shonkoff et al., 2021). These disparities are evidenced early with significant gaps in school readiness scores among some minority race children, many of whom are immigrants (Ghandour et al., 2019).

In this context, early childhood services hold great potential for RIC. These programs can reduce the impact of early life disparities by intervening during sensitive periods of development when children are most responsive to interventions. Early childhood services can build protective factors, address the impacts of traumatic experiences, promote school readiness in RIC, boost social-emotional development, and connect parents with comprehensive resources and supports, including exposure to new social networks (Lee et al., 2018; Mondi et al., 2021; Neitzel, 2020; Saint Gilles & Carlson, 2020; Vesely et al., 2013). Decades of literature have clarified the compensatory power of high-quality early childhood services for reducing disparities in achievement, health, and well-being in children experiencing early life disadvantage and stress exposure (Negussie et al., 2019; Phillips & Shonkoff, 2000). Indeed, the power of these programs, when comprehensive, high-quality, and occurring very early in life, appears to hold the greatest return of investment over any other intervention point in the lifespan (García et al., 2016). In the USA, subsidized child care and early education (CCEE), including Head Start and state-funded preschool; maternal, infant, and early childhood home visiting; and IDEA Part C early intervention and Part B preschool special education, represent widely accessible and publicly funded early childhood services with evidence of impact on far-reaching outcomes for children and families.

### Child Care and Early Education

Participation in high-quality early care and education programs, including Head Start and state-funded preschool in the USA, has been associated with wide-ranging benefits for children, ranging from short-term improvements in school readiness to long-term improvements in achievement and health and reductions in criminal activity (Amadon et al., 2022; Campbell et al., 2012; Gormley et al., 2023; Negussie et al., 2019; Schoch et al., 2023). ECE programs can also help to counteract the negative effects of trauma on children’s well-being and development (Saint Gilles & Carlson, 2020). High-quality early childhood education services from birth to age 5 are thought to generate close to a 14% return on investment in downstream costs (García et al., 2016). Evidence of effectiveness has been demonstrated in the children of immigrants, with greater attendance in Head Start and public pre-k and associated improvements in school readiness scores (Lee et al., 2018).

### Maternal, Infant, and Early Childhood Home Visiting

Home visiting has been associated with significant impacts on a wide range of maternal and child outcomes (Avellar & Supplee, 2013; Filene et al., 2013), including reduced incidence of low birth weight, health problems, emergency department visits, and child welfare involvement and enhanced child development, cognitive abilities, and reductions in child behavioral concerns (Michalopoulos et al., 2019; Peacock et al., 2013). In addition, these programs have been demonstrated as effective in reducing parent depression, anxiety, and stress (Goldfeld et al., 2021; Roberti et al., 2022; Vismara et al., 2020) and have been associated with reductions in family reliance on public benefits over time (Olds et al., 2010). Some home visiting models have been implemented in refugee and immigrant communities with evidence of effectiveness. For example, the Baby TALK model was associated with improvements in child socioemotional and language development, parental stress, and parental trauma symptoms (Hilado et al., 2018). The program additionally supported the economic self-sufficiency of enrolled families and supported their access to other needed services and supports.

### Early Intervention

IDEA Part C Early Intervention services provide critical support to children with developmental delays and disabilities to enhance development and minimize the need for additional, more costly intervention later in development (National Early Childhood Technical Assistance Center [NECTA], 2011). Access to high-quality early intervention, especially early on, can remediate and shift developmental trajectories, leading to less utilization of special education supports later on (NECTA, 2011; Ullery & Katz, 2016) and improvements in functional skills (McManus et al., 2019). Families additionally find early intervention to be impactful, improve their capacity to meet and advocate for their child’s needs, and enhance their child’s behavioral, social, and developmental skills (Bailey et al., 2005; Guralnick, 2011; Hebbeler et al., 2007).

### Underutilization of Early Childhood Services by Refugee and Immigrant Families

Despite the benefits of early childhood services and the demonstrated needs of RIC, newcomer communities are found to be underrepresented in early childhood programs in the USA (Gallegos et al., 2021; Park & Katsiaficas, 2019; Van Lancker & Pavolini, 2023). Refugee and immigrant families, despite being broadly eligible, are less likely to enroll in child care (Greenberg et al., 2019), Head Start (Hardy et al., 2020), early intervention (Gallegos et al., 2021), and home visiting (Park & Katsiaficas, 2019). There is little data available on how widely immigrant and refugee children are reached; however, children that speak minority languages and children of first- or second-generation immigrants are significantly less likely to access early intervention services (United States Government Accountability Office, 2023).

### Barriers to Access

The underrepresentation of newcomer families in early childhood services may be due to a lack of awareness of potential services and their benefits, parental preferences and cultural beliefs about early childhood, fear and mistrust of formal or governmental institutions, difficulty navigating complex systems, and a limited supply of services, including services that are culturally adapted.

Families’ perceptions of early childhood services are shaped by cultural beliefs (e.g., traditional gender norms), past experiences, and stigma (Beatson et al., 2022). The perspectives and preferences immigrant parents hold about early care and education may not align with the perspectives of early childhood educators in the USA (Tobin & Kurban, 2018). Families with fear or concern related to immigration status may also hesitate to enroll in formal child care programs (Karoly & Gonzalez, 2011); this can also be exacerbated by changing federal enforcement and immigration policies (Matthews et al., 2018). Changing government priorities may also drive program eligibility changes, as in the present administration of the USA where efforts are underway to actively limit access for undocumented children (U.S. Department of Health and Human Services, 2025). These concerns may be compounded for refugee families with previous experiences of betrayal, persecution, and mistreatment by government entities who may distrust programs (Signorelli et al., 2017). Finally, families may be reluctant to seek out early intervention services due to stigma around developmental delays and difficulties, exacerbated in some cases by cultural beliefs around disability (Kang-Yi et al., 2018; Signorelli et al., 2017).

Though there is evidence of these preferences and hesitancy around engaging with formal early care and education institutions, research has suggested that the gap in services cannot be entirely explained by parental preferences (Karoly & Gonzalez, 2011). Underutilization of these services by refugee and immigrant families may be related to lack of awareness of services or to the complexity of early childhood systems in the USA. Newcomer parents may not be aware of the free or subsidized services available to them, the processes for enrolling, and the benefits of these services to their children and whole families (Hanson & Espinosa, 2016; Karoly & Gonzalez, 2011; Seibel, 2021). These barriers are exacerbated by the fact that information about early childhood services are often only available in the host country’s primary language and that refugees and immigrants may not have access to social and professional networks that share information about available services when they first migrate (Brown et al., 2020).

The composition of communities and newcomer arrivals is dynamic and indicates a growing need for services that are culturally responsive. Between 2003 and 2011, the USA welcomed refugees from over 100 countries who spoke almost 300 different languages (Capps & Newland, 2015). Despite this immense diversity, language services and materials related to early childhood service offerings may only be available in a few select languages. Language-related barriers can impact family awareness of and ability to access early childhood programs (Sykes, 2018). In addition, screening tools, such as validated early childhood development screeners, may not be translated into less common languages spoken by local newcomer communities, resulting in difficulties accurately identifying potential developmental delays and disabilities (Bevan et al., 2020). Families’ comfort level with the providers and services available may also impact their enrollment and participation in these services.

### Theoretical Framework

Access to early intervention and other developmentally enriching resources and supports can reduce, limit, or even close the impacts of early adversity experienced by young children in families who may have limited resources or capital (Duncan & Sojourner, 2013; Phillips & Shonkoff, 2000; Votruba‐Drzal et al., 2004) and whose parents may be managing the weight of their own unmet needs and historic or ongoing adversity and trauma (Herba et al., 2013; Miles et al., 2019; Steele et al., 2016). In this way, high-quality early childhood interventions can promote equity for children who may have uneven opportunities early in life (García et al., 2016), due to the brain’s sensitivity to high-quality environments and intervention during the first 5 years of life (Shonkoff, 2011). However, the very children these interventions are designed to benefit may have greater challenges accessing them. Structural barriers frequently identified in families experiencing poverty, such as difficulty securing reliable transportation, unpredictable shift work, and unstable employment (Haider, 2021), may intersect with barriers that are specific to the immigrant and refugee experience and the cultural capital required to successfully navigate the early childhood system in the USA. Cultural capital refers to the knowledge, attitudes, styles, and qualifications obtained via socialization and education that signal intelligence in a particular cultural context and which enable social mobility (Bourdieu, 1986). Greater cultural capital has been associated with participation in early care and education programs (Ripamonti, 2023), and, in newcomer families, the absence of cultural capital during acculturation may lead to less or different forms of engagement with systems and institutions (Baquedano-López et al., 2013), presenting an additional barrier that compounds with other structural and immigrant-specific barriers. Thus, without interventions that can address the accumulation of hardships and the intersections of identities and experiences of newcomer families, children in immigrant households face complex barriers to participation in early childhood programs. These barriers prevent equitable access to the experiences that can contribute to the cultural capital, social mobility, well-being, and achievement of RIC.

As global migration continues to expand, programs and agencies are in need of strategies to effectively address barriers and increase equitable access to early childhood services for newcomer families. Understanding the strategies associated with access to early childhood services can also help policymakers and service providers understand where best to target their efforts. However, there is limited understanding of the strategies being implemented to improve enrollment and participation in early childhood programs among refugee and immigrant populations. In addition, parent preferences and the degree to which preferences align with strategies being implemented are currently unclear. This study addresses this gap by conducting a scoping review of research on strategies being implemented to increase newcomer families’ participation in services and parent preferences for early childhood services. The review has two research questions:

1. What strategies have been implemented or proposed by programs to promote newcomer family access to early childhood programs?
2. How are newcomer parent preferences related to participation in early childhood services?

## Methods

As a scoping review, this study aims to systematically identify and map the available evidence on a topic, including the type of available research, key factors related to the topic or concept, and gaps in the available knowledge base (Arksey & O’Malley, 2005; Munn et al., 2018). A scoping review was selected for this study due to the small body of work available to date, the diverse research methods utilized among published papers, and limited reviews on the topic (for one exception, see Brown et al., 2020, although they do not include a focus on home visiting and early intervention). This review follows Arksey and O’Malley’s (2005) five-step scoping review framework including identifying the research question; identifying relevant studies; selecting studies; charting the data; and collating, summarizing, and reporting the results. The study additionally utilized the Preferred Reporting Items for Systematic Reviews and Meta-analyses Extension for Scoping Reviews (PRISMA-ScR) checklist (Tricco et al., 2018).

### Identification and Selection of Studies

Searches were conducted in four academic databases: PsycInfo, PubMed, SocINDEX, and Web of Science, and supplemented with five website searches for grey literature: the Urban Institute, AIR, Child Trends, Mathematica, and Migration Policy Institute. Database searches were conducted using combinations of identified key words (depending on the search engine’s limitations) that included identification of refugee OR immigrant and an array of early childhood services. For example, the following terms were used to search PsychInfo:

(refugee OR immigrant OR “unaccompanied minor” OR asylee OR “temporary protected status” OR “victims of trafficking” OR “trafficked victims” OR T-Visa OR U-visa OR Cuban OR Haitian OR Amerasian) AND (evaluation OR impact OR program OR intervention OR policy OR Project OR train OR training OR review OR meta-analysis OR synthesis) AND (“early childhood program” OR “early childhood services” OR “early childhood development” OR “early care” OR “early education” OR “child care” OR “early learning” OR “preschool” OR “Early Head Start” OR “Head Start” OR “home visit” OR “home visits” OR “home visiting” OR “comprehensive services” OR “two-generation” OR “whole family” OR “family resource center” OR “early intervention”).

Websites with grey literature were searched using individual key terms, available filters, and topics or tags, per website specifications. For example, the following filters were applied, and search terms were entered individually to identify unique articles on the Migration Policy Institute website:

Filters: 1/1/2013–present, research publications, “early childhood education and care” topic area, “children and family policy” topic area.

Search terms: “early childhood program,” “early childhood services,”“early childhood development,”“early care,”“early education,”“child care,”“early learning,”“preschool,” “Early Head Start,”“Head Start,”“home visit,”“home visiting,”“comprehensive services,”“two-generation,”“whole family,”“family resource center,” “early intervention”.

**Fig. 1:**
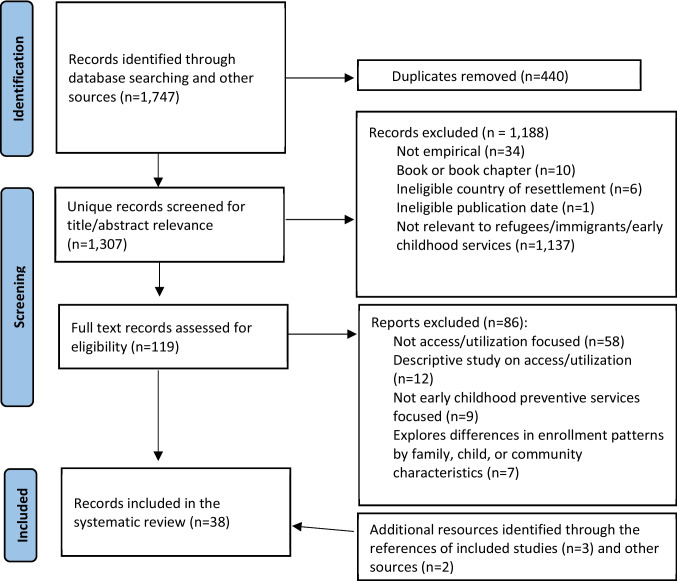
PRISMA of search, identification, screening, and inclusion process. Note: Page MJ, et al. BMJ 2021;372:n71. https://doi.org/10.1136/bmj.n71; this work is licensed under CC BY 4.0. To view a copy of this license, visit https://creativecommons.org/licenses/by/4.0/

A full set of search terms for each database and website can be provided by the authors upon request. Searches were conducted between November and December 2023, and the search results yielded 1307 unique results across databases and websites (see Fig. 1).

### Inclusion and Exclusion

Studies needed to meet inclusion criteria presented to be eligible for this review (see Table 1). All included studies examined strategies supporting RIC access to early childhood services or parent perspectives on desired services. Early childhood services included those with federal and state public funding, often subsidizing access for children in households with low incomes. These programs either do not assess eligibility on the basis of citizenship or immigration status or, in some instances, they may require parent proof of residency or other verification as determined by state policies. Titles and abstracts were screened for empirical studies published in English between 2013 and 2023 and with relevance to immigrants’ and refugees’ access to early childhood services (see Table 2). One hundred and nineteen full-text manuscripts were reviewed. The lead researcher and two coders triple-coded 16 full text manuscripts independently, discussing any discrepancies and clarifying the application of criteria, until agreement was established. Any other disagreements were discussed and resolved by the lead researcher. Studies were excluded at this stage if they did not have a focus on refugees or immigrants, early childhood services, and strategies to enhance access to or parent preferences for early childhood services. For example, studies that described the prevalence of disparities or barriers that RIC face to access but did not report on strategies or approaches to reduce those disparities or barriers were excluded. Following full text review, 33 articles were selected for inclusion (see Fig. 1). An additional five manuscripts were identified in the references of reviewed articles and other web-based sources. A final set of 38 manuscripts met eligibility criteria and were coded.

**Table 1:** Article inclusion and exclusion criteria

Inclusion	Exclusion
Empirical studies	Book and book chapters, opinion pieces, and implementation studies
Organisation for Economic Co-operation and Development (OECD) countries	Countries not listed as OECD countries were not included
Paper was written or available in English	Paper not written or unavailable in English translation
Paper was published between 2013 and 2023	Published prior to 2013
Study population is immigrants and/or refugees or study population is focused on providing services to refugees/immigrants	Not focused on immigrants and/or refugees or includes immigrants and/or refugees within a larger aggregated sample that includes nonimmigrants
Focus on publicly funded early childhood services including child care, Head Start/Early Head Start, preschool, home visiting, early intervention, or 2 generation programs	Not focused on publicly funded early childhood services
Focus on access and utilization of early childhood services, including strategies to promote access and parent preferences that inform access or utilization	Not focused on strategies or preferences related to the promotion of access or utilization. May provide descriptive information (e.g., rates of enrollment), focus exclusively on barriers to access, or focus on differences in enrollment as stratified by family, child, or community characteristics

**Table 2:** Overview of included studies and study information

Author(s)	Year	Method	Sample size	Population	Region of origin	Host country	Strategy	Preferences
Child care								
Albesher*	2022	Qualitative	14	Refugees	Middle East, Africa, Asia	USA	x	
Drange and Telle	2015	Quantitative	23,707	Immigrants	Multiple	Norway	x	
Fakhari et al	2023	Qualitative	14	Immigrants	Multiple	Canada	x	
Fede*	2018	Quantitative	4709	Immigrants	Multiple	USA	x	
Ferreira van Leer and Coley	2023	Mixed methods	744	Immigrants	Latin America	USA		x
Galarza-Heras**	2014	Quantitative	100	Immigrants	Mexico	USA		x
Lin*	2020	Quantitative	269,511; 8640	Immigrants	Multiple	USA	x	
Sandstrom and Gelatt	2017	Mixed methods	~ 2720	Immigrants	Multiple	USA		x
Tobin	2020	Qualitative	~ 300	Immigrants	Multiple	Multiple		x
Preschool and Head Start								
Adams et al.**	2016	Mixed methods	Not reported	Immigrants	Multiple	USA	x	
Ansari	2017	Quantitative	5850	Immigrants	Central America, South America	USA		x
Ansari et al	2020	Qualitative	30	Immigrant	Central America	USA	x	x
Chan	2020	Quantitative	3100	Immigrants	Multiple	USA	x	
Choi*	2022	Qualitative	6	Immigrants	Korea	USA		x
Gelatt et al.**	2014	Qualitative	~ 40	Immigrants	Multiple	USA	x	
Greenberg et al.**	2018	Mixed methods	240	Immigrants	Multiple	USA	x	x
Greenfader and Miller	2014	Quantitative	1141	Immigrants	Multiple	USA	x	
Jang	2020	Qualitative	8	Immigrants	Korea	Canada		x
Morland et al.**	2016	Qualitative	33	Refugees	Multiple	USA	x	
Vesely	2013	Qualitative	40	Mixed	Africa, Latin America	USA	x	x
Multiple early childhood care and service settings								
Eastern*	2022	Qualitative	4	Refugees	South Asia, Middle East	USA		x
Estes et al.**	2022	Mixed methods	125; 25	Immigrants	Multiple	USA	x	
Gross and Ntagengwa**	2016	Qualitative	Not reported	Refugees	Multiple	USA	x	
Johnson et al	2017	Quantitative	1050	Immigrants	Multiple	USA		x
Miller et al	2013	Quantitative	2500	Immigrants	Multiple	USA		x
Miller et al	2014	Quantitative	10,400	Immigrants	Multiple	USA		x
Park and McHugh**^,1^	2014	Mixed methods	~ 70	Mixed	Multiple	USA	x	
Park et al.**^,1^	2018	Qualitative	Not reported	Mixed	Multiple	Multiple	x	
Poureslami et al	2013	Qualitative	119	Mixed	Asia, Middle East	Canada		x
Ressler et al	2020	Quantitative	6400	Immigrants	Mexico	USA		x
Satkowski et al	2016	Qualitative	278	Mixed	Central America, South America	USA		x
Shuey and Leventhal^2^	2020	Qualitative	256	Immigrants	Central America	USA		x
Swenson and Hawes**	2020	Qualitative	24	Mixed	Africa, Middle East	USA	x	x
Vesely et al	2021	Qualitative	55	Immigrants	Central America	USA		x
Early intervention								
Hurley et al	2014	Qualitative	28	Refugees	Multiple	USA	x	
Woolfenden et al	2015	Qualitative	40	Mixed^3^	Multiple	Australia	x	
Home visiting								
Jean-Baptiste et al	2017	Qualitative	81	Immigrants	Multiple	USA	x	
Wickramasinghe et al.	2020	Mixed methods	5	Refugee	Middle East	UK	x	

Child care includes the use of any nonparental child care, including center and home-based care; sample size may include both providers, parents, and refugee or immigrant children

^*^The study is a dissertation

^**^The study is found in the grey literature

^1^One of the service settings relates to home visiting

^2^One of the service settings relates to early intervention; mixed setting studies typically include combinations of programs including Head Start, preschool, and child care

^3^Study sample is defined as “culturally and linguistically diverse”

### Charting the Data

**Fig. 2:**
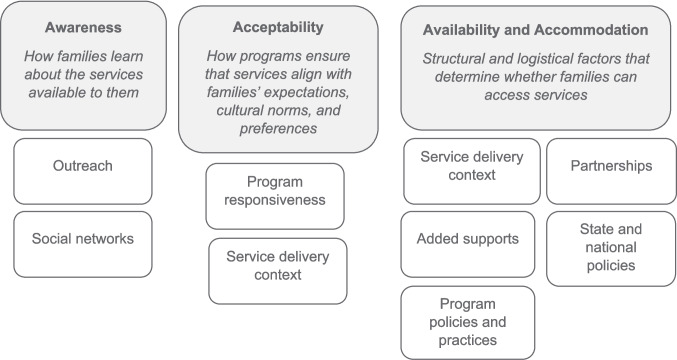
Strategies to promote access to early childhood services. Note: Strategy groupings were adapted from Archambault et al.’s Framework of Access to Quality Early Childhood Education and Care (ECEC) for Children from Disadvantaged Backgrounds (2020)

Strategies were coded for sample size, research methodology, population of interest (refugees, immigrants, or both), country or region of origin, host country, and early childhood services studies. Coded strategies included initiative and program features described in the article methods, program design, and results. Strategy coding was informed by Archambault et al.’s Framework of Access to Quality Early Childhood Education and Care (ECEC) for Children from Disadvantaged Backgrounds (2020), which identifies five broad buckets of features impacting supply-side access to early care and education such as acceptability, availability and accommodation, affordability, and appropriateness. These buckets were modified to fit the focus of this review, ultimately including awareness, acceptability, availability, and accommodation as overarching categories (see Fig. 2). Within these categories, coders identified sub-categories as they emerged in articles, applying them to all articles. Examples of subcategories included outreach, program policies, and program responsiveness (see Table 3). Seventy-five percent of articles were double-coded, and areas of disagreements were resolved through discussion, along with input from the lead researcher, prior to establishing a final code structure. Finally, the connection of strategies to enrollment or access-related outcomes was coded when described within papers (see supplemental materials). Parental preferences were coded using a similar strategy. Two coders noted key themes as they emerged and used them to create a broad set of codes which were then applied to the full set of parent preference studies (see supplemental materials). The lead researcher double-coded 30% of articles focused on parent preferences, resolving any identified discrepancies through discussion, prior to establishing a final code structure that was applied to the included studies.

**Table 3:** Strategies described to reduce barriers and enhance access to early childhood services by study

Study information	Strategies									Outcomes		
Author and year	Awareness		Acceptability		Availability and accommodation					Trust	Negative perceptions	Enrollment/utilization
	Outreach	Social networks	Workforce responsiveness	Program responsiveness	Service delivery context	State and national policies	Program policies	Partnerships	Added supports			
	57%	43%	57%	67%	52%	29%	43%	43%	52%			
Child care												
Drange and Telle (2015)	x			x	x	x			x	—	—	**↑**
Fede (2018)						x				—	—	**↑**
Lin (2020)						x	x			—	—	**↑**
Albesher (2022)	x	x								—	—	**—**
Fakhari et al. (2023)	x	x	x	x						—	—	**—**
Preschool and Head Start												
Adams et al. (2016)	x	x	x	x	x		x	x	x	—	—	**—**
Ansari et al. (2020)	x	x		x	x			x	x	—	↓	**↑**
Chan (2020)			x			x	x			—	—	**↑**
Gelatt et al. (2014)	x	x	x	x	x	x	x	x	x	—	—	**—**
Greenberg et al. (2018)	x	x	x	x	x	x	x	x	x	↑	—	**↑**
Greenfader and Miller (2014)							x		x	—	—	**↑**
Morland et al. (2016)	x	x	x	x	x		x	x	x	—	—	**↑**
Vesely (2013)	x	x								—	—	**—**
Multiple early childhood care and service settings												
Estes et al. (2022)			x	x	x		x	x	x	—	—	**—**
Gross and Ntagengwa (2016)	x		x	x	x			x		—	—	**—**
Park and McHugh (2014)	**x**		**x**	**x**			**x**	**x**		**—**	**—**	**—**
Park et al. (2018)												
Swenson and Hawes (2020)				**x**						**—**	**—**	**—**
Early intervention												
Hurley et al. (2014)				**x**						**—**	**—**	**—**
Woolfenden et al. (2015)	**x**	**x**	**x**	**x**	**x**				**x**	**—**	**—**	**—**
Home visiting												
Jean-Baptiste et al. (2017)			**x**		**x**				**x**	**↑**	**↓**	**↑**
Wickramasinghe et al. (2020)			**x**	**x**	**x**			**x**	**x**	**—**	**↓**	**↑**

## Results

Of the 38 articles, 11 (29%) were quantitative research, 20 (53%) were qualitative research, and an additional 7 (18%) used mixed methods (see Table 2). Thirty studies (79%) were conducted in the USA, and the majority of studies investigated samples of immigrants (*n* = 25, 66%), followed by refugees (*n* = 6, 16%) or both immigrants and refugees (*n* = 7, 18%). Typically, studies included immigrants and refugees from multiple regions of origin (76%). Most studies focused on strategies or parent preferences related to mixed early childhood services (e.g., child care and preschool, *n* = 14, 37%), preschool including Head Start (*n* = 11, 30%), or child care (*n* = 9, 24%). In contrast, early intervention and home visiting were addressed separately in only two studies each (5% each). Twenty-two articles included strategies relating to access or utilization of early childhood services among refugee and immigrant samples (Table 3), and 20 articles addressed parent preferences related to accessing ECE (Table 4).

**Table 4:** Parent preferences related to early childhood services by study

Study information	Methods	Preferences				
Author and year		Academic and socioemotional development	Type of care	Perceived quality	Cultural responsivity and match	Language
		80%	65%	65%	60%	50%
Child care						
Galarza-Heras (2018)	Quant	x	x	x	x	x
Sandstrom and Gelatt et al. (2017)	Quant	X	x	x		
Tobin (2020)	Qual	x			x	x
Ferreira Van Leer and Coley (2023)	Mixed	x	x	x		x
Preschool						
Ansari (2017)	Quant	x	x		x	
Ansari et al. (2020)	Qual	x		x		x
Choi (2021)	Qual	x			x	x
Greenberg et al. (2018)	Qual	x		x		x
Vesely (2013)	Qual	x	x		x	x
Jang (2020)	Qual				x	x
Multiple settings or services						
Johnson et al. (2017)	Quant	x	x		x	x
Ressler et al. (2020)	Quant	x		x		
Eastern (2022)	Qual	x	x	x	x	
Poureslami et al. (2013)	Qual	x			x	
Miller et al. (2013)	Quant		x	x	x	
Miller et al. (2014)	Quant		x	x	x	
Satkowski et al. (2016)	Qual		x	x		
Shuey and Leventhal (2020)	Qual	x	x	x		
Swenson and Hawes (2020)	Qual	x	x	x	x	
Vesely et al. (2021)	Qual	x	x	x		x

### Strategies for Increasing Access or Addressing Barriers to Access

A variety of strategies were identified in included papers within the broader strategy groupings of awareness, acceptability, and availability and accommodation (see Fig. 2).

#### Awareness

Strategies that increase parents’ awareness of the range of early childhood rely on outreach through networks accessed by families to share information and bolster positive perceptions of the early childhood services available in communities. Fifty-nine percent of papers discussed strategies supporting awareness related to *outreach*, including utilizing language-accessible materials, parent or community liaisons, conducting outreach through providers and organizations that already serve families, and outreach directly to families to share information about available services. An additional 45% of papers discussed strategies supporting awareness related to *social networks* as a method by which parents leverage their connections to learn about early childhood programs and navigate enrollment.

##### Outreach

Families may learn about early childhood programs through service providers and community-based organizations (CBOs) that families already interact with, such as healthcare providers and social workers (Adams et al., 2016; Ansari et al., 2020; Fakhari et al., 2023; Gelatt et al., 2014; Morland et al., 2016; Park & McHugh, 2014; Vesely, 2013). For example, families learn about and are referred to Head Start programs through a robust referral network in Massachusetts comprised of healthcare providers and CBOs (Park & McHugh, 2014).

##### Social Networks

Families’ use of their social networks was frequently raised as a means through which parents may learn about early childhood services in a new country (Albesher, 2022; Ansari et al., 2020; Fakhari et al., 2023; Gelatt et al., 2014; Greenberg et al., 2018; Woolfenden et al., 2015; Vesely, 2013). Parents may leverage their social networks, including family and friends, to learn about early childhood development generally and to gather information on early childhood systems and programs (Ansari et al., 2020; Wickramasinghe et al., 2020). In one study, close to three quarters of mothers relied on social connections to identify child care and early education arrangements, and, in many cases, social connections also supported mothers directly with the procurement and completion of enrollment paperwork (Vesely, 2013). Families may have a greater sense of trust when their personal network shares about programs in their native language and in culturally appropriate ways (Fakhari et al., 2023; Gelatt et al., 2014). In one study, participants in three states indicated that “word of mouth” was essential in supporting preschool enrollment (Greenberg et al., 2018). However, it is notable that robust social networks may not be readily available and accessible to all newcomer families. Families who have arrived more recently in the country may not yet have a well-established social network (Ansari et al., 2020; Greenberg et al., 2018), and isolation may be especially salient in families without legal documentation (Vesely et al., 2021). Some families speaking less common languages may not have a social network and may feel “alienated and unwelcome” in the programs they are enrolled in (Park & McHugh, 2014). Some strategies appear to hold the dual focus of supporting parents’ social networks while increasing access to services such as early intervention screening. For example, free and supported playgroups and parent groups in Australia were noted as a source of social support and a strategy to promote access to early intervention (Woolfenden et al., 2015). Although parents in the study frequently experienced social isolation, mothers described playgroups as “life savers” (Woolfenden et al., 2015). Similarly, playgroups were noted as a gateway to early childhood program participation in another study, as they helped increase parents’ comfort with other caregivers (Park et al., 2018).

#### Acceptability

Strategies that increase the acceptability of early childhood services for parents bridge across cultural backgrounds, past experiences, and needs to support parents’ sense of inclusion. Sixty-eight percent of studies discussed strategies supporting acceptability related to *program responsiveness*, which includes providing language resources to families, hiring interpreters, facilitating parent involvement, implementing trauma-informed practices, and incorporating culturally responsive practices into program implementation. An additional 59% of papers discussed themes supporting acceptability related to the *workforce responsiveness* of early childhood programs, highlighting practices such as hiring culturally and linguistically matched staff and training staff with the aim of increasing cultural competency.

##### Program Responsiveness

Programs can implement a series of practices to increase family understanding and comfort with service activities and goals. A variety of strategies exist to support language access, especially when linguistically matched staff are unavailable. Programs have hired staff specifically to provide interpretation or utilized phone-based interpretation services (Gelatt et al., 2014; Greenberg et al., 2018; Hurley et al., 2014; Morland et al., 2016; Park & McHugh, 2014; Wickramasinghe et al., 2020; Woolfenden et al., 2015). A Migrant Head Start program utilized simplified and clear language in outreach materials distributed to ESL families to enhance understanding among families with varied levels of literacy (Gelatt et al., 2014). In addition, some programs have co-located English courses or other professional development-focused courses on-site to engage parents alongside children (Ansari et al., 2020; Drange & Telle, 2015; Estes et al., 2022; Gelatt et al., 2014; Morland et al., 2016; Park et al., 2018). Indeed, engaging parents intentionally appears to be a core function of programs that successfully serve immigrant families (Park & McHugh, 2014).

Programs were described as taking a number of steps to include parents and create opportunities for their involvement within early childhood programs (Ansari et al., 2020; Gelatt et al., 2014; Greenberg et al., 2018; Gross & Ntagengwa, 2016; Park & McHugh, 2014; Wickramasinghe et al., 2020). For example, preschool programs have aimed to create a welcoming environment and open channels of communication with families by hosting facility tours and conducting home visits between teaching staff and families, actions which may increase parent trust and ultimately boost enrollment (Greenberg et al., 2018). Establishing trust between programs and families was identified as pivotal for successfully discussing early childhood development concerns in one study (Woolfenden et al., 2015), with strategies noted such as maintaining a fluid, open-ended style of interaction that either encouraged parents to identify issues on their own or that involved sensitively raising issues directly.

##### Workforce Responsiveness

Programs can develop their workforce through hiring culturally and linguistically matched staff and preparing program personnel to enhance their cultural competence. Multiple studies raise the importance of hiring culturally and linguistically diverse staff that match the characteristics of the communities served or hiring interpreters when the staff could not be directly hired (Gelatt et al., 2014; Greenberg et al., 2018; Gross & Ntagengwa, 2016; Jean-Baptiste et al., 2017; Morland et al., 2016; Park et al., 2018; Woolfenden et al., 2015). In some instances, efforts to hire from the community can require that programs adapt processes and bolster their available supports to ensure the workforce can meet necessary qualifications. These adaptations can be resource-intensive, such as establishing a nontraditional workforce pipeline with additional training support (Greenberg et al., 2018). One Head Start and refugee resettlement agency partnership resulted in the hiring of up to ten refugees as case workers and translators who supported placing refugee children in high-quality center-based child care (Gross & Ntagengwa, 2016). Another Migrant Head Start program trained their staff to become qualified as instructors, allowing them to train and credential parents from the community as early childhood educators (Gelatt et al., 2014). Hiring culturally and linguistically matched staff, such as other immigrant parents, may be an important way to build trust with families. Such a workforce was identified as promoting parents’ initial sense of safety with early care and education (Fakhari et al., 2023) and influential in supporting family access to early intervention services (Woolfenden et al., 2015).

When hiring from the community is not feasible, feeling respect from early childhood professionals has been identified as essential (Swenson & Hawes, 2020; Woolfenden et al., 2015). Programs can provide training to staff on a range of topics, including cultural competence, the cultural needs of the community being served, migration experiences, working with dual language learner families, and trauma-informed care as strategies to increase staff cultural responsiveness (Adams et al., 2016; Gross & Ntagengwa, 2016; Morland et al., 2016; Wickramasinghe et al., 2020).

#### Availability and Accommodation

Strategies that accommodate families’ needs and promote the availability of services address the logistical and structural barriers that may impede families from participation. Fifty-five percent of studies discussed strategies supporting accommodation related to the *service delivery context* of programs such as providing home-based services, hosting services in public spaces, and co-locating multiple services. An additional 55% of papers discussed strategies supporting accommodation related to providing *added supports* to families, such as providing free meals during the day or transportation. Forty-five percent of studies discussed strategies supporting accommodation and availability related to *program policies and practices* and *partnerships*, respectively, highlighting program and agency policies that could enhance the accommodation of newcomer family needs and partnerships with other CBOs, agencies, and intersectoral efforts to support access. Finally, 27% of studies discussed strategies supporting the availability of care through *state and national level policies* that impact access to services, including generous subsidy policies and expanded universal child care.

##### Service Delivery Context

Community-embedded approaches were frequently deployed in studies aiming to overcome barriers to access. For example, some studies described the co-location of services or the strategic placement of services in settings where families are comfortable or frequently attend (Estes et al., 2022; Greenberg et al., 2018; Morland et al., 2016; Park et al., 2018). One program in Australia organized early childhood services in schools that children attended or shopping centers, reporting an increase of more than 45% in initial appointment attendance following referral (Wickramasinghe et al., 2020). Home visiting programs that engage with families in the locations where they feel most comfortable, such as their homes or other community-based settings, may increase trust (Jean-Baptiste et al., 2017; Wickramasinghe et al., 2020). These programs similarly have the potential to reach families who are vulnerable due to isolation and limited knowledge of other available services (Park et al., 2018). The placement of programs in strategic locations where high concentrations of newcomer families reside may also effectively promote access. A publicly funded child care program in Norway focused on neighborhoods with high populations of immigrant families (Drange & Telle, 2015), while a preschool in Texas was placed in an area where more than half of families identified as Latinx or as English language learners, with the intent to reduce barriers to access (Ansari et al., 2020).

##### Added Supports

Instrumental supports, such as providing transportation, enrollment support, and warm referrals, may be important levers to overcome some barriers (Ansari et al., 2020; Jean-Baptiste et al., 2017; Gelatt et al., 2014; Greenberg et al., 2018; Morland et al., 2016). A state-funded preschool program in Texas developed a bus route to transport children from preschool to local schools, where some parents had their older children enrolled. This step enabled participation by allowing parents to drop off and pick up multiple children enrolled in different programs at the same time and location (Ansari et al., 2020). In one qualitative study of MIECHV home visitors serving a high proportion of immigrant mothers, home visitors described that driving enrolled mothers to needed appointments, including the hospital, had supported immigrant mothers’ retention and participation (Jean-Baptiste et al., 2017). Enrollment-specific supports may also support parents with managing the complexity of navigating enrollment for early care and education programs. Some studies identified strategies to provide such support including translating enrollment forms and hosting enrollment events with hands-on support in locations where families feel comfortable (Gelatt et al., 2014; Greenberg et al., 2018; Morland et al., 2016). One study described the potential of “welcome centers” administered by school districts to facilitate enrollment in school-based and referrals to other essential services for newcomers (Park et al., 2018).

##### Program Policies and Practices

Several papers discussed program-specific policies that could enhance access, such as prioritizing refugees on waiting lists or modifying enrollment requirements (Gelatt et al., 2014; Greenberg et al., 2018; Morland et al., 2016), presumably by overriding first-come first-serve approaches which favor better-resourced families. An analysis of the nationally representative Head Start Impact Study found that randomized lottery selection into Head Start led to the elimination of disparities between enrollment in children of immigrants and nonimmigrants (Greenfader & Miller, 2014).

Other program policies reduce barriers by including fewer or more flexible documentation requirements. For example, Head Start programs without income requirements or that accept dual-language learners regardless of family income were identified as easiest to access (Gelatt et al., 2014). In programs where documentation was required, programs implemented flexible policies that allowed a wide range of documents, including self-certification in some cases, to verify income (Gelatt et al., 2014). In addition, programs that intentionally collect less documentation on families may reduce their apprehension related to participation (Estes et al., 2022). Program practices that maximalize flexibility for the employment-schedule needs of families, such as by offering flexible or extended hours of attendance, may reduce barriers (Gelatt et al., 2014; Greenberg et al., 2018; Park et al., 2018). For example, a refugee resettlement agency in Arizona co-located and supplemented a half-day Early Head Start and Head Start program with child care services to effectively extend the hours of care and enable working parents to participate (Morland et al., 2016).

##### Partnerships

Collaboration and partnership between organizations, including between early childhood service providers and resettlement agencies, may effectively reduce barriers to access (Adams et al., 2016; Gelatt et al., 2014; Greenberg et al., 2018; Gross & Ntagengwa, 2016; Morland et al., 2016; Park & McHugh, 2014; Park et al., 2018; Vesely, 2013; Wickramasinghe et al., 2020). Collaboration between Head Start and refugee resettlement agencies in Arizona, New York, and Texas resulted in increased enrollment into Early Head Start and Head Start programs in refugee communities (Gross & Ntagengwa, 2016; Morland et al., 2016). Key features of these collaborations attributed to increased enrollment included “cross-trainings” about each other’s services, a jointly developed case management system, and joint outreach to families (Morland et al., 2016). Partnerships between early childhood programs and community-based organizations (CBOs) or healthcare organizations that serve RIC may also be an important mechanism for enhanced access (Adams et al., 2016; Gelatt et al., 2014; Greenberg et al., 2019; Morland et al., 2016; Park & McHugh, 2014; Vesely, 2013; Wickramasinghe et al., 2020). For example, partnerships may facilitate cross-agency and cross-sector knowledge of programs and services in the community and enhance outreach efforts.

##### State and National Policies

Policies can be effective in reducing barriers and systemically facilitating access to early care and education services among refugee and immigrant families (Chan, 2020; Drange & Telle, 2015; Fede, 2018; Gelatt et al., 2014; Greenberg et al., 2018; Lin, 2020). Two papers highlighted the impacts of free universal early childhood programs. A natural experiment conducted in Norway demonstrated that access to community-wide free child care increased the enrollment of immigrant families (Drange & Telle, 2015). Families in five city districts with high populations of immigrant families were offered 20 h of free child care per week through a program that included an active recruitment approach toward families with eligible children, a curriculum tailored to the needs of children in immigrant families, and optional language classes during child care hours to encourage parent engagement. Immigrant children in “intervention” districts had a 12% increase in enrollment and demonstrated significantly higher test scores at school entry than children residing in communities without the hours of free care (Drange & Telle, 2015). In Illinois, a policy implemented in 2010 required public school districts to identify dual-language learning children and provide them with access to bilingual classrooms in which teachers were certified in bilingual education or English as a second language along with early childhood education (Chan, 2020). An analysis of data from before and after this policy went into effect demonstrated that the policy resulted in an 18–20% increase in enrollment among dual-language learner children.

In the USA, state-level implementation of Child Care and Development Fund (CCDF) policies may have an impact on low-income immigrant families’ likelihood of enrollment into center-based child care (Lin, 2020). Factors include the generosity of the subsidy policies including the income eligibility threshold set by each state, continuing income eligibility, family copayment rates, base provider reimbursement rates, and the relative ease of application process, including the verification of parents’ identification, verification of children’s citizenship status, and the length of the time before redetermination of eligibility. States with more generous initial income eligibility thresholds, no requirement to verify child citizenship or immigration status, and a 12-month re-determination period were found to have higher rates of center-based child care enrollment among immigrant children (Lin, 2020). Other policies may have an indirect effect on family access to child care. One analysis examined the effect of policies allowing undocumented immigrants access to drivers’ licenses in New Mexico, Utah, Washington, and California (Fede, 2018). In the states where this policy went into effect (New Mexico, Utah, and Washington), enrollment in early care and education among households with at least one undocumented adult family member appeared to increase among 3–5-year-old children, whereas in California, where the policy passed but did not go into effect, participation rates in these households were unchanged.

### Parent Preferences

In addition to studies exploring strategies for promoting access, this systematic review captured studies exploring parental preferences relating to early childhood services. Though parental preferences differ significantly between communities and individual families, it is important to build an understanding of parameters around which parents search for and evaluate early childhood programs. Of the 37 studies included in the systematic review, 20 studies discussed parental preferences. Twelve studies (60%) explored parent preferences qualitatively, using methods such as case studies, interviews, and focus groups to elicit priorities from parents through discussion; seven (35%) used quantitative methods to measure the impact of certain preferences on enrollment or participation, and one used mixed methods to first explore dimensions important to parents and then measure their impacts on enrollment. All of these studies included preferences discussed in the context of child care, Head Start, or preschool; no parental preference studies discussed home visiting or early intervention services. Five overarching themes emerged from papers that discussed parental preferences for early childhood programs: academic and socioemotional development, variation in preference around types of care, perceived quality of the program and providers, the importance of cultural respect and responsivity, and variation in preferences around the language of the program. See Table 4 for an overview of the prevalence of preferences across studies. These preferences, among others, impact parents’ likelihood of enrolling their child in an early childhood education program and the type of care they are likely to select.

#### Academic and Social-Emotional Skills

Multiple parents across studies discussed academic and socioemotional development as motivating factors for enrolling their children in early childhood programs (Ansari et al., 2020; Eastern, 2022; Greenberg et al., 2018; Poureslami et al., 2013; Swenson & Hawes, 2020). These preferences were identified in both qualitative and quantitative studies (Sandstrom & Gelatt, 2017). Parents differed in whether they expected early childhood programs to prioritize social development (Choi, 2021; Poureslami et al., 2013), school readiness skills (Jang, 2020), or cognitive abilities (Poureslami et al., 2013). Though these factors were often discussed broadly, parents shared nuanced expectations for the role of early childhood programs in academic and socioemotional development. Some parents felt early childhood education was crucial to supporting socioemotional development, especially as their children adapted to a new culture and community (Poureslami et al., 2013). Preference for the promotion of social skills can increase the likelihood that parents enroll in formal early childhood programs (Galarza-Heras, 2018); however, other parents held the perspective that social and behavioral skills should be taught within the home (Poureslami et al., 2013). Another study found that parents sought programs that included educational activities but did not necessarily prioritize academic preparation for later schooling (Shuey & Leventhal, 2020). Some parents worried that limited English proficiency and recent migration put their child at a disadvantage when entering school and saw early childhood programs as an opportunity to remediate that gap (Tobin, 2020). In two studies, parents who prioritized educational components of programs and the development of school readiness skills were more likely to enroll their children in Head Start and other formal child care and education programs (Johnson et al., 2017; Ressler et al., 2020).

#### Type of Care

Parents across studies varied in their preferences for types of care. Multiple studies found that, when possible, parents preferred maternal care or care provided by family members, especially for infants and toddlers (Eastern, 2022; Ferreira Van Leer & Coley, 2023; Swenson & Hawes, 2020; Vesely et al., 2021). One study found this preference was shaped by parents’ belief in the importance of children spending time with their parents and siblings and lack of trust for caretakers outside of the family unit to provide appropriate care (Shuey & Leventhal, 2020); however, these parents were more comfortable enrolling their children in Head Start, when necessary, in comparison with informal care settings (Shuey & Leventhal, 2020). Vesely and colleagues (2021) similarly found that mothers were more comfortable with center-based care than informal neighbor care, if parental or other known caregivers were not available. This preference was informed by the importance they placed on licensed care, which signaled the provider’s trustworthiness (Vesely et al., 2021). Child age and ability to communicate about their experiences also informed parents’ comfort level with care outside of the home (Vesely et al., 2021). Other studies found that parents were motivated to enroll their children in center-based care or other formal ECE because of their perception that it offered a strong early learning environment and social-emotional development (Jang, 2020; Poureslami et al., 2013; Vesely, 2013). Immigration factors such as level of English proficiency, time since immigration, and country of origin also inform preferences for types of care (Sandstrom & Gelatt, 2017); immigrants with limited English proficiency, immigrant families from Mexico, and more recent immigrants were less likely to consider center-based care when evaluating their child care options (Sandstrom & Gelatt, 2017).

#### Perceived Quality

Parents alluded to the importance of the quality of available care through discussions on safety, provider characteristics, and program efforts to engage parents. Parents discussed the importance of safety measures such as gates and locked doors and expressed concern over the treatment of their children in programs (Ansari et al., 2020). In multiple studies, parents described seeking caregivers who were trustworthy, caring, nurturing, and who were well prepared to care for children (Swenson & Hawes, 2020; Ferreria Van Leer & Coley, 2023; Sandstrom & Gelatt, 2017). They sometimes noted the importance of preparation to support children with trauma-informed approaches (Greenberg et al., 2018). Parents also discussed the quality of programs in terms of their efforts to welcome, engage, and communicate with parents, efforts which also contributed to parents’ comfort with providers (Choi, 2021; Galarza-Heras, 2018; Greenberg et al., 2018; Satkowski et al., 2016). Program flexibility was also identified as an important care feature to parents (Ansari, 2017; Sandstrom & Gelatt, 2017). In some studies, parents’ preference for care that they consider to be high quality increased the likelihood that they enrolled in center-based care (Miller et al., 2014; Satkowski et al., 2016).

#### Cultural Respect and Responsivity

Across studies, parents often highlighted the importance of their culture being respected and reflected in their child’s program. Parents in some studies discussed specific considerations such as culturally relevant food, relevant holidays and celebrations, and other efforts to integrate language and culture directly into programming (Choi, 2021; Eastern, 2022; Poureslami et al., 2013). For example, parents highlighted the importance of nutritious meals that accommodated cultural restrictions or norms (Choi, 2021; Eastern, 2022). Parents emphasized that having an educator in the program that matched their cultural background helped facilitate their child’s transition to school, cultural understanding, and communication between the program and home environment (Jang, 2020; Vesely, 2013). Quantitative studies have found that preferences for cultural consistency, including the caregiver and family sharing the same race or ethnicity (Johnson et al., 2017), increased the likelihood that families would enroll in programs such as preschool, home-based child care, and Head Start (Ansari, 2017; Johnson et al., 2017; Miller et al., 2013). Other parents discussed cultural responsiveness more broadly, indicating that they sought a program that would acknowledge and respect their culture (Poureslami et al., 2013; Swenson & Hawes, 2020) and support their family fitting into the program community. For example, parents did not wish to stand out as the only immigrant family or family from their cultural background (Vesely, 2013). Indeed, parent preferences related to cultural sensitivity are associated with an increased likelihood of enrollment in formal ECEs (Galarza-Heras, 2018).

#### Languages of the Program

Although preferences were divided, parents consistently raised the importance of provider language across studies. Some studies found that parents preferred to enroll their children in programs where staff speak the same language as the family or where children have opportunities to speak and practice their native language (Choi, 2021; Tobin, 2020). Vesely and colleagues (2021) found that Latina immigrant mothers prioritized finding a child care program where staff spoke the family’s native language, as this allowed mothers to engage meaningfully in their child’s education, such as by helping with assignments. Other parents, in contrast, prefer that programs focus on English language development (Greenberg et al., 2018). Jang (2020) found that Korean immigrant parents selected an English-only program instead of a bilingual option because they wanted their children to have exposure to English immersion, knowing that their children would have continued exposure to Korean at home. Preferences around language were also informed by parents’ perceptions of the resources available within their community. One study found that parents who lived in areas with a small Spanish-speaking population were more likely to seek a bilingual ECE program, whereas those in communities with larger Spanish-speaking populations prioritized programs that would support their child’s English fluency (Tobin, 2020).

## Discussion

This scoping review synthesizes the literature on strategies that may support refugee and immigrant family access to early childhood services. While some studies described the implementation of these strategies via interventions that enhanced access for refugee or immigrant families (e.g., Drange & Telle, 2015), others qualitatively described provider perspectives on what had worked or what was needed (e.g., Hurley et al., 2014), and still others addressed parent preferences for early childhood services (e.g., Ferreira Van Leer & Coley, 2023). This scoping review searched for articles across publicly funded early childhood services, including child care and early education, home visiting, and early intervention, seeking to characterize early evidence and perspectives on what can work within and across these systems to effectively reduce widespread disparities in access. Broadly, strategies associated with enhanced access typically organized around increasing family awareness of programs, enhancing the acceptability of programs to culturally and linguistically diverse families, and implementing policy and program-level strategies that systemically change the availability and accommodation of services. Several key themes emerged across these strategies.

The centrality of trusted relationships was a consistent and prominent feature, a finding corroborated in other reviews focused on supporting access in this population (Archambault et al., 2020; Brown et al., 2020). Professional and personal networks were frequently described as precursors and drivers of access. These relationships likely support families with managing the challenges of acculturation (Brown et al., 2020), which are more stressful in culturally disparate host countries from the newcomer’s country of origin (Demes & Geeraert, 2014). In addition, trusted relationships may hold an influential role in remediating lost or damaged institutional trust prior to migration (Essex et al., 2022). In one study of Syrian and Iraqi refugee and asylum seekers, traumatic experiences in their countries of origin were related to lower trust in governmental institutions and, unexpectedly, to higher trust in social connections (Hall & Werner, 2022). Thus, it may be that trusted relationships in the host country, along with increasingly positive experiences, serve to gradually restore family trust in institutions (Essex et al., 2022), which may include early childhood programs. These relationships may also serve to build cultural capital during acculturation (Baquedano-López et al., 2013) and aid parents in addressing additional intersecting barriers that prevent their participation.

Early childhood providers’ use of trauma-informed approaches may be one instrumental way in which trust can be built among newcomer families (Burgund Isakov & Markovic, 2024). Such approaches require that providers have knowledge of the cultural needs of families, an understanding of the traumatic experiences and stressors that families have experienced across migration contexts (including during resettlement or other early arrival processes), and the awareness of triggers and risks for re-traumatization (Burgund Isakov & Markovic, 2024; Miles et al., 2019). Training and development that equips early childhood professionals to provide trauma-informed care may promote the formation of trusting relationships with families and may ultimately increase child participation in early childhood programs. Trust is also recognized as a critical component of child care search and selection even beyond immigrant populations (Sandstrom et al., 2024).

A subset of strategies focused on increasing the adaptations within programs to increase cultural responsiveness toward families. A range of studies described programmatic efforts to hire and train providers that were representative of the communities being served. When hiring local staff was not possible, some studies described other efforts such as orientation trainings focused on cultural competence and the pairing of interventionists with interpreters (Wickramasinghe et al., 2020). These data also mirror parents’ preferences, prioritizing shared cultural background with early childhood educators or efforts by the program to respect and acknowledge their families’ diverse backgrounds. Although parent perspectives on the importance of linguistic match were split, several studies demonstrate how the diversity of the early childhood workforce is impactful, with fewer enrollment-related disparities as access to linguistically diverse early childhood educators increases (Johnson et al., 2017; Lin, 2020; Miller et al., 2014).

The strategies with the most robust evidence of impact come from several quasi-experimental quantitative studies examining the effectiveness of policies in increasing child care and early education enrollment among immigrant children. These policies were implemented in some communities or states and not others (e.g., Drange & Telle, 2015) and at discrete points in time (Chan, 2020), allowing comparison of how enrollment rates shifted on a population level. These strategies coalesce in their shared focus on creating universally accessible care opportunities for all children, as opposed to providing subsidized care to a select few. In addition, these policies often intentionally focus on identifying, outreaching, and meeting the unique learning needs of bilingual children. Such approaches address systemic barriers that intersect with other family barriers, such as by requiring programs to identify and appropriately serve bilingual learners (Chan, 2020) and allowing undocumented parents access to drivers’ licenses (Fede, 2018).

Several recently implemented policies in the European Union similarly aim to enhance access to child care and early education for immigrant children by ensuring free child care, removing requirements that parents provide evidence of their residence and income, and in some cases targeting undocumented children (De Koster, 2023). Although evaluations of these policies are forthcoming, these policies reflect that solutions to systemic disparities are likely most effective when targeted toward the supply side of care; that is, the way systems and programs are designed to reach families (Archambault et al., 2020). Policies implemented at scale such as these hold the potential to influence outcomes and access to opportunities for the largest number of newcomer children. Such strategies require significant investment and commitment of resources, which likely hold implications for the feasibility and sustainability of enacting such policies. For instance, developing universal access for newcomer families to culturally appropriate early childhood services likely requires the layering of additional strategies such as developing a workforce pipeline to ensure a qualified workforce (e.g., Gelatt et al., 2014), the placement of programs in locations heavily populated by newcomer families (e.g., Drange & Telle, 2015), translation and adaptation of materials, and infrastructure to support training and assessments of quality. Thus, the successful implementation of some strategies may require a substantial and coordinated initial public investment, in contrast to strategies that may be feasible for individual programs to implement without significant prior planning or resources.

Finally, many strategies focused on practices, supports, and intentional design features that reduce structural barriers preventing families’ participation. For example, one clinic-based early childhood nurse program was adapted to be flexibly implemented in community-based settings. These changes led to more than a 40% increase in successful referrals to the program (Wickramasinghe et al., 2020). Other programs have focused on offering instrumental supports to families, such as providing transportation between care programs and schools (Ansari et al., 2020) and extending a half-day preschool program for working parents by coordinating the provision of additional child care programming on site (Morland et al., 2016). Altogether, as strategies build cultural capital for families and reduce systematic and structural barriers, families may experience fewer intersecting barriers to accessing early childhood programs for their children.

Some analyses find that immigrant and US-born parents share preferences related to child care, although their search behaviors may vary (Sandstrom & Gelatt, 2017), and it may be that some care selection differences relate to the type of care available. Analyses have demonstrated a lower concentration of early care and education options, including Head Start, available in immigrant communities (Hardy et al., 2020; Xing, 2019), suggesting that supply problems contribute to the barriers faced by families. Thus, efforts to actively reduce barriers experienced by immigrant and refugee families represent an important lever for equity.

### Limitations

Most of the available evidence in the peer-reviewed and grey literature addressed access to child care and early education, including Head Start and state-funded preschool, whereas strategies to promote access among culturally and linguistically diverse families to home visiting and early intervention had very little peer-reviewed literature in our search, with only two articles each focusing exclusively on these service systems. It may be that additional evidence on home visiting, early intervention, and other early childhood programs exists in the international literature under specific programmatic names that were not captured in this review due to the use of general search terms and only US-specific programs. For example, our search terms included the US-specific Head Start program, but terms specific to other country programs were not included.

The absence of studies on home visiting and early intervention are notable, as these critical services support long-term well-being and can remediate early developmental delays (Correll et al., 2023; Filene et al., 2013; National Early Childhood Technical Assistance Center, 2011). Young children of refugees have often experienced trauma, including family separation across migration (Miles et al., 2019), and the children of immigrants in mixed-status families have systematically experienced widespread trauma following the implementation of policies that allowed the involuntary and extended separation of young children from their undocumented parents (Dreby, 2015). These experiences have been associated with attachment disruptions and cascading academic and developmental concerns in children (Casasnovas, 2022) and likely impact many of the more than 5 million children in the USA with at least one undocumented parent (Capps et al., 2016). Thus, this gap in available evidence is especially urgent and in need of research attention. Future research should seek to describe rates of participation in early intervention and home visiting among newcomer families, including how participation may vary by host country, family country of origin, and length of time since arrival. Research should also characterize how IDEA part C and Part B-619 programs Child Find processes in the USA are actively searching for and identifying children in immigrant and refugee families prior to compulsory schooling age. Children below the age of six face gaps in services that have been noted as being invisible on a policy level due to a fundamental lack of responsibility and disconnect between the services of early childhood programs and resettlement services (Park et al., 2018). Studies should be conducted on the successful strategies utilized to increase access to early intervention and home visiting, including efforts to culturally adapt and embed these programs into newcomer communities.

The quality of the evidence for included papers was not assessed and reported in this review. This limitation is related to the largely qualitative and correlational body of work available on this topic. Many of the strategies described in this review are framed in diverse ways across papers with varied levels of depth. Some strategies are only mentioned briefly as part of a program’s approach to reach immigrant and refugee families, while others are described at length and in direct relation to outcomes such as enrollment. In addition, outcomes are not consistently or quantitatively measured across studies; rather, they are qualitatively described in some papers, and the links between strategies and outcomes are not always made explicit. Thus, our thematic analysis of strategies is suggestive and in need of additional empirical research for further validation.

Most reviewed articles did not include content on the feasibility or sustainability of deployed strategies to support access. As evidence grows on the described strategies in this review, research studies are needed to initially document their implementation feasibility, as well as the cost and mechanisms utilized to sustain their implementation. Such information will be critically useful for policymakers and program leaders selecting among strategies to pilot.

While this paper assesses the evidence on policy, program, and provider strategies to enhance access, other factors on the family and child level may also be linked with differences in enrollment and utilization of early childhood services. For example, parental support for early learning and academic skills with their young children is positively associated with preschool enrollment (Ansari & Crosnoe, 2015); higher parent education, employment, and socioeconomic status have been linked with greater enrollment (Miller et al., 2013, 2014; Shuey & Leventhal, 2020); and studies demonstrate that older children are more likely to be enrolled in child care and preschool programs (Ansari, 2017; Shuey & Leventhal, 2020). However, very little is known about family, child, or community factors associated with utilization of early childhood services outside of child care and early education. Finally, many papers in our sample did not differentiate refugees from other immigrants or mixed-status families, limiting insight into how strategy effectiveness might vary by subgroup. Future studies should attempt to disentangle whether and how strategies differentially support access for subgroups of newcomer families.

## Implications and Conclusion

This review examines solutions to the pervasive access issue faced by the children of refugees and immigrants across most major early childhood services. Indeed, while the barriers to access are increasingly characterized and understood, less is known about how to reduce these disparities. This review synthesizes the array of strategies being deployed to reach families in accessible ways, adapt services to meet their needs, overcome barriers, and address issues of supply. The strongest evidence reviewed indicates that enrollment in child care and early education can be influenced by state, national, and program policies that combat disadvantages based on family language, background, and geographic location and direct additional resources toward immigrant and refugee communities. Other findings present a series of immediately actionable promising approaches, supplemented by parent perspectives on preferences for child care and early education. More research is needed on the success of these approaches and how they may vary by subpopulations (e.g., refugees, immigrants with lawful status, undocumented immigrants) and by countries of origin. Ultimately, reducing disparities in child access holds the potential to promote the well-being of the next generation. By strengthening families, remediating early delays, nurturing positive child development, and preparing children for school, early childhood services have the potential to set a strong foundation for all children following migration.

## Supplementary Information

Below is the link to the electronic supplementary material.

ESM 1 (23.0 KB DOCX)

## Data Availability

All data used to support these findings were extracted from the included studies which are referenced throughout the article and in the reference list.
