# Supplementary material for: A Scoping Review of Strategies to Increase Newcomer Family Access to Early Childhood Services
Source: Prev Sci. 2026 Feb 11;27(2):185–207. doi: 10.1007/s11121-025-01867-y (PMC12999706; doi:10.1007/s11121-025-01867-y)
Supplement: Supplementary file 1 — (23.0 KB DOCX) [file 11121_2025_1867_MOESM1_ESM.docx]

**Supplemental Materials**

**Appendix A. Detailed Search Approach**

For academic databases, the research team used Boolean Phrases or advanced search functions to combine multiple search terms (see below for an example). They also manually restricted results to the years of 2013-2023 and searched terms within the titles and abstracts of articles. The research team used database retrieval guidance (Gusenbauer & Haddaway, 2018) to inform the initial development of search term strings that were best aligned with the functionality of each database, resulting in slightly varied search terms combinations by database. This strategy was utilized for PsycInfo, PubMed, SocIndex, and Web of Science.

The team also conducted manual searches of relevant research and policy organization websites. They selected relevant focus areas within websites or search engines, limited results to research publications published between 2013 and 2023, and searched individual key terms until they reached saturation of results. The key terms searched on each website varied by the focus area of each organization. For example, on the Migration Policy Institute website, the team searched for relevant articles using only key terms related to early childhood programs, whereas on the Urban Institute website, the team searched using key terms related to immigrants or refugees and early childhood programs. This search strategy was used for the American Institute for Research (AIR), Child Trends, Mathematica, Migration Policy Institute, Research Connections, and the Urban Institute.

**Example search terms**

| Academic databases | Research and policy organization websites |
| --- | --- |
| (refugee OR immigrant OR “unaccompanied minor” OR asylee OR “temporary protected status” OR “victims of human trafficking” OR “trafficked victims” OR T-Visa OR Cuban OR Haitian OR Amerasian) AND (evaluation OR impact OR program OR intervention OR policy OR project OR review OR meta-analysis OR synthesis) AND (“early childhood program” OR “early childhood services” OR “early childhood development OR “early care” OR “home visit” OR “comprehensive services” OR “two-generation” OR “whole family” OR “family resource center” OR “early intervention”) | Refugee  Immigrant  Early childhood services  Early childhood development  Child care  Home Visiting  Early Intervention |

**Appendix B. Strategy to Increase Access Codebook**

**Inclusion and exclusion:** The strategies table presented in the manuscript was created by coding studies that discussed strategies for increasing access or utilization of early childhood services for refugee and immigrant families using the following codebook. Both peer-reviewed and non-peer-reviewed studies were coded in this table. Strategies cited in the introduction or literature review sections of a paper were not included. Strategies that are suggested by the authors, usually in the discussion section, are not included. Two codes may reflect aspects of the same strategy.

**Codebook:** The codebook reflects subcodes underneath the main codes through letters. All subcodes were collapsed to “x”s to simplify the interpretability of the table in the manuscript (i.e. if any subcode was present in an article, the final table will reflect an x under the main code for that study).

| **Construct** | **Codes** | **Definition/Description** |
| --- | --- | --- |
| **Awareness** | **Outreach** | |
|  | L = language-accessible materials | Program provides outreach materials in languages spoken by families. |
|  | C = parent or community liaisons | Explicitly recruiting other parents or trusted members of the community for outreach efforts. |
|  | P = outreach through providers/organizations that already serve families | Programs note that parents have learned about their services through community organizations (or materials posted at community organizations). This would also include instances when early childhood programs host outreach and enrollment events at resettlement, CBO, or health organizations |
|  | F = outreach directly to parents | This code captures efforts to inform parents about ECE, services available to them, or how to access those services/ This code also captures strategies that mention outreach to families, but don’t necessarily indicate the specifics of the outreach |
|  | **Social** | |
|  | N = social networks | Paper discusses how families/parents interact and engage with people within their social networks (e.g. relatives, neighbors) to learn more about enrolling/accessing programs. |
|  | I = interventions to improve social integration | Programs have activities for families to participate in that are intended to build their social network or work toward social integration. |
| **Acceptability** | **Workforce**  **Responsiveness** | |
|  | H = employing providers from multicultural backgrounds | Program employs staff from multicultural backgrounds (which could include staff who are immigrant/refugees themselves) |
|  | T = training staff on cultural sensitivity/interacting with refugee and immigrant families | Includes certification for bilingual teachers, includes professional development opportunities for staff |
|  | W = well-qualified staff | Having well-qualified staff on the program team. |
|  | **Program Responsiveness** | |
|  | C = generic cultural sensitivity/responsiveness | Program mentions cultural responsivity but does not describe its practices in detail. Meant to capture that programs are thinking about/prioritizing cultural sensitivity when we don’t have enough details to code for specific components or we have multiple strategies that fit under this umbrella but not under the other defined codes (add a + if this is the case). |
|  | P = facilitating parent involvement | Program makes efforts to involve parents through ongoing activities.    Program allows parents in the classroom/to engage directly in services with their children |
|  | R = trauma-informed practices | Programs use trauma-informed practices, staff are trained in trauma-informed responses |
|  | D = engage families in program design | Parents and families are engaged throughout the design of the program to ensure responsiveness to their needs |
|  | A = additional programs or courses for parents | Program offers various courses or trainings for parents to engage in. |
|  | I = services available to support families with language | Any type of service or support that is available to families that supports language access. This could be interpretation services, translators, phone interpreters. |
| **Availability and Accommodation** | **Service Delivery** | |
|  | C = co-located with other services | Program provides multiple services in one location (eg: healthcare screenings and playgroup) (early childhood program does not have the be the provider of these other services) |
|  | G = geographically tailored | Services/program is focused specifically on neighborhoods or locations where families are based or in response to their needs. Program may also be located in a place that is responsive to the needs/preferences of newcomer communities. |
|  | H = programs are offered at home | Services/programs are offered at family’s home. |
|  | P = programs are offered in public spaces | Services are offered in areas that are publicly accessible by families. |
|  | **National and state policies** | |
|  | N = national policy | National policy enacted that expands access to early childhood services |
|  | S = state policy | State policy enacted that expands access to early childhood services |
|  | **Program policies** | |
|  | E = changes to  eligibility requirements/verification | Anything that a program or policy does to provide more flexibility or reduce burden to the family during the program enrollment process. Note that this is distinct from “enrollment supports”, which are supports programs may provide to facilitate the enrollment process for a family, such as translating materials, having walk-in hours, etc. |
|  | R = random assignment | This code indicates that children were randomly assigned access to an early childhood program |
|  | D = data collection | At a broader level, any mention of how data collection efforts can be modified or adapted to better meet the needs of immigrant and refugee populations, or at a more focused level, considering which data should be collected with awareness of how this impacts immigrant/refugee families. |
|  | H = flexible hours of care | Program provides extended or adapted hours of care |
|  | S = accommodate siblings | Programs adjust programming to accommodate siblings in some way |
|  | **Partnerships** | |
|  | C= community-based organizations | Study mentions partnership or collaboration with CBOs or community members/leaders broadly |
|  | H = healthcare | Study mentions partnership or collaboration with health care providers |
|  | R = resettlement agencies | Study mentions partnership or collaboration with resettlement agencies or resettlement agency staff |
|  | A = intersectoral/interagency collaboration | Study mentions intersectoral collaboration between early childhood agency, resettlement agency, or other agencies. |
|  | **Added supports** | |
|  | M = material supports | Includes any material supports, such as cost (program is free to families), food, or transportation. |
|  | W = warm referrals | Study mentions warm referrals as part of the program’s approach |
|  | E = enrollment supports | Program has staff or supports available to parents to specifically support with enrollment into early childhood or other programs. |

**Appendix C. Preferences Codebook**

**Inclusion and exclusion:** Studies that discussed parent preferences and perceptions relating to early childhood services were coded using the following codebook. The preferences table presented in the manuscript also includes quantitative studies that measured the impact of parents holding certain preferences around child care on their likelihood to enroll in care. Constructs were coded if they were identified by the study as being important to parents’ search for care, parental preferences around care, or perceptions of the care parents currently use for their children.

**Codebook:**

| Codes | Description |
| --- | --- |
| Type of care | Differential preferences for types of care (i.e. parental care, family, friend or neighbor (FFN) care, center-based care, home-based care, etc.  Quantitative studies that link parent preferences to the use of different care types. |
| Language | Differential preferences around language learning in early childhood program (i.e. bilingual vs. English-only) |
| Cultural respect and responsivity | Preferences for programs that respect and include families' culture, programs whose staff or program “matches” their culture, etc. (e.g. cultural activities, providers with similar backgrounds, culturally relevant food) |
| Perceived quality | Preferences for “quality” of care which included explicit discussions of quality, or discussions around aspects of programs that contribute to its quality (i.e. trust, safety, comfort, class sizes, parent involvement/engagement, flexibility, quality of caretakers, preparation and training of caretakers) |
| Academic and socioemotional skills | Differential preferences for the emphasis placed on academic, cognitive, socioemotional, or a combination of these in early childhood programs. |
